# Effects of water flossing on gingival inflammation and supragingival plaque microbiota: a 12-week randomized controlled trial

**DOI:** 10.1007/s00784-023-05081-4

**Published:** 2023-05-25

**Authors:** Xin Xu, Yishan Zhou, Chengcheng Liu, Lei Zhao, Ling Zhang, Haolai Li, Yi Li, Xingqun Cheng

**Affiliations:** 1 The State Key Laboratory of Oral Diseases & National Clinical Research Center for Oral Diseases, West China Hospital of Stomatology, Sichuan University, Chengdu, Sichuan China; 2 Department of Cariology and Endodontics, West China Hospital of Stomatology, Sichuan University, Chengdu, Sichuan China; 3 Department of Periodontology, West China Hospital of Stomatology, Sichuan University, Chengdu, Sichuan China; 4 State Institute of Drug/Medical Device Clinical Trial, West China Hospital of Stomatology, Sichuan University, Chengdu, Sichuan China; 5 Bixdo (SH) Healthcare Technology Co., Ltd., Shanghai, China; 6 Department of Geriatric Dentistry, West China Hospital of Stomatology, Sichuan University, No. 14, 3rd section of Renmin South Road, Chengdu, 610041 Sichuan China

**Keywords:** Dental plaque, Gingivitis, Halitosis, Oral microbiota, Water flossing

## Abstract

**Objectives:**

The effects of water flossing on dental plaque removal have been suggested, but its ecological impact on dental plaque microbiota needs further investigation. In addition, whether this plaque control measure by water flossing promotes the control of halitosis still needs clinical validation. The aim of this study was to evaluate the effects of water flossing on gingival inflammation and supragingival plaque microbiota.

**Materials and methods:**

Seventy participants with gingivitis were randomly assigned to control (toothbrushing) and experimental (toothbrushing + water flossing) groups (*n* = 35). Participants were recalled at 4, 8, and 12 weeks, and their gingival index, sulcus bleeding index, bleeding on probing, dental plaque index, and oral malodor values were measured. The microbiota of supragingival plaque was further investigated using 16S rRNA sequencing and qPCR.

**Results:**

Sixty-three participants completed all revisits (control: *n* = 33; experimental: *n* = 30). The experimental and control groups exhibited similar clinical characteristics and dental plaque microbiota at baseline. Adjunctive water flossing effectively reduced the gingival index and sulcus bleeding index as compared to the toothbrushing control group. The water-flossing group showed reduced oral malodor at week 12 as compared to the baseline. Consistently, the water-flossing group exhibited altered dental plaque microbiota at week 12, characterized by a depletion of *Prevotella* at genus level and *Prevotella intermedia* at species level as compared to the toothbrushing control. In addition, the plaque microbiota of water-flossing group exhibited a more aerobic phenotype, while the control group was more anaerobic.

**Conclusions:**

Daily water flossing can effectively alleviate gingival inflammation and reduce oral malodor, possibly by depleting oral anaerobes and altering the oral microbiota to a more aerobic phenotype.

**Clinical relevance:**

Water flossing adjunctive to toothbrushing effectively alleviated gingival inflammation, representing a promising oral hygiene practice to promote oral health.

**Clinical trial registration:**

The trial was registered in the Chinese Clinical Trial Registry (http://www.chictr.org.cn/showprojen.aspx?proj=61797, #ChiCTR2000038508) on September 23, 2020.

**Supplementary Information:**

The online version contains supplementary material available at 10.1007/s00784-023-05081-4.

## Introduction

The periodontal diseases are highly prevalent and can affect up to 90% of the worldwide population [1]. Gingivitis is the most prevalent form of periodontal disease. According to the released data from the fourth national oral health epidemiology survey of China, the prevalence of gingival bleeding is over 87.4% in middle-aged and elderly people in China [2]. Gingivitis is typically caused by dental plaque accumulation. *Streptococcus*, *Fusobacterium*, *Actinomyces*, *Veillonella*, and *Treponema*, and possibly *Bacteroides*, *Capnocytophaga*, and *Eikenella*, are etiologically involved [3]. Microbial colonization and participation is sequential, with the complexity of the associated flora increasing with time [3]. Other local or systemic etiologic factors such as defective prosthesis, smoking, and hormones may also promote plaque deposition and/or predispose the host to microbial attack [4]. The ecological balance via both inter-microbial and host-microbial interactions plays a critical role in maintaining the gingival tissue health [5]. Disruption of this homeostatic balance leads to selective outgrowth of species with potential for destructive inflammation, and increases in the bacterial burden promote gingival inflammation in this condition [5, 6]. Gingivitis is a key risk factor of periodontitis, and control of gingival inflammation is crucial for the primary prevention of periodontitis [7]. In addition, halitosis, which shares risk factors with periodontal diseases, is also common among patients with gingivitis or periodontitis [8].

Dental plaque control, typically by toothbrushing, is the most effective measure for preventing and treating gingivitis [9]. However, toothbrushing leaves approximately 40% of the dental plaque on the tooth surface [10]. A toothbrush combined with interdental cleaning device can achieve sufficient oral hygiene [10, 11]. Regular flossing can remove up to 80% of the interproximal plaques as reported by American Dental Association [12]. Daily use of dental floss can effectively reduce the gingival inflammation and halitosis [13, 14]. However, regular flossing requires time and skill, limiting its daily use [15]. Interdental brushes can effectively reduce the dental plaque between the teeth, and alleviate the gingival inflammation [16, 17]. However, interdental brushes can only be used if there is sufficient space between the teeth, and we should choose the appropriate diameters; otherwise, the cleaning efficiency will be influenced and the periodontal tissue may be destroyed [17, 18].

The power-driven water flosser, with claims of easy home use, has become a widely recommended oral hygiene product. Through pulsation and pressure, it disrupts plaque and removes loosely lodged debris, and can deliver antimicrobial solutions into the sulcus and interproximal regions [19]. The effects of water flossing on dental plaque removal and gingivitis management were better than those of regular floss and interdental brushes [20, 21]. Water flossing adjunctive to manual toothbrushing can inhibit dental plaque formation and reduce the levels of pro-inflammatory factors, alleviating gingivitis and reducing periodontitis recurrence as compared to solely toothbrushing [22-24]. In addition, water flossing can alleviate gingivitis in orthodontic patients and manage peri-implant mucositis [25, 26]. With the widespread acceptance and application of water flossing, the safety regarding its daily use has drawn increasing attention. Studies have demonstrated that daily use of water flosser for 3 months did not increase the risk of bacteremia during periodontal maintenance therapy [27, 28]. In addition, water flossing was safe to be used on composite restorations with no influence on their surface roughness and color stability [29].

However, water flossing cannot completely eradicate dental plaque due to microbial recolonization on the tooth surface shortly after cleaning [30]. Long-term disturbance of oral microbiota by measures such as antimicrobial mouthwash (chlorhexidine) may have the risk of microbial dysbiosis and bacterial resistance [31, 32]. Therefore, the long-term effect of water flossing on oral microbial ecology still needs clinical validation. In addition, whether this plaque control measure by water flossing promotes the control of halitosis, which is a common complaint among patients with gingivitis, still needs investigation. Here, we conducted a 12-week prospective clinical trial to investigate the effect of water flossing on gingival inflammation and bleeding, plaque accumulation, and halitosis in individuals with gingivitis, and the ecological impact of water flossing on oral microbiota was further evaluated.

## Materials and methods

### Study participants

This article is reported according to CONSORT guidelines for reporting randomized clinical trials. This study was approved by the Research Ethics Committee of West China Hospital of Stomatology (WCHSIRB-D-2020-309) and was in agreement with the Declaration of Helsinki and complied with Chinese Good Clinical Practice (GCP) regulations. All participants were recruited at the West China Hospital of Stomatology, Sichuan University, and signed informed consent. The trial was registered in the Chinese Clinical Trial Registry (http://www.chictr.org.cn/showprojen.aspx?proj=61797, #ChiCTR2000038508) on September 23, 2020.

The participants were aged 18–65 years with no systemic diseases. All participants had intact Ramfjord teeth (16, 21, 24, 36, 41, 44) in the oral cavity. Participants had gingivitis, with a modified gingival index ≥ 1, dental plaque index ≥ 1.5, and gingival sulcus depth < 3 mm [33]. Participants were excluded if they had periodontitis, had undergone surgical/non-surgical periodontal therapy or antibacterial/hormonal drug therapy in the last 6 weeks, had participated in other clinical trials within the last 3 months, had an allergic constitution, or were pregnant [34].

### Experimental design

**Fig. 1:**
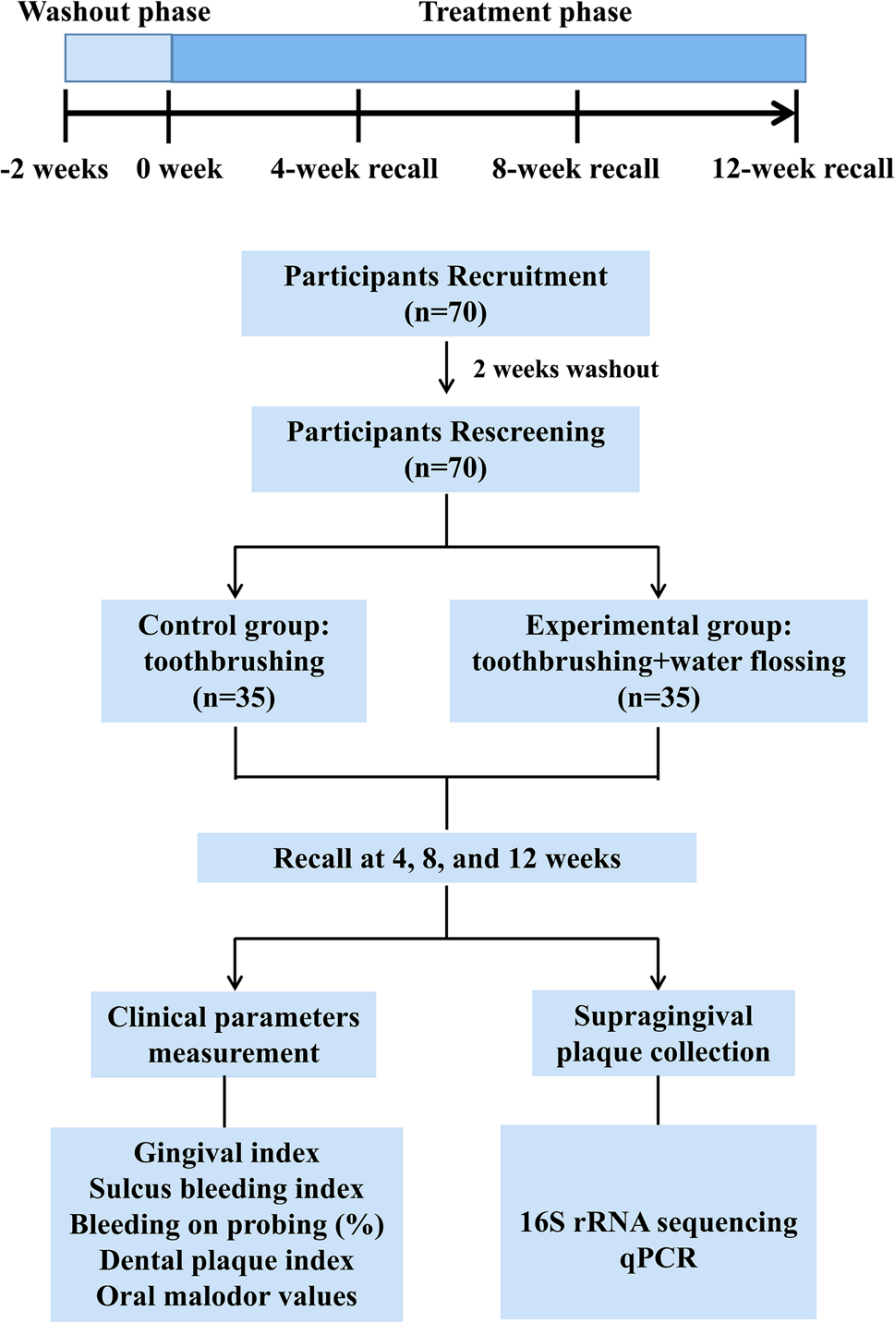
Schematic of study design

This was a single-center, randomized controlled clinical trial with a duration of 14 weeks (2-week washout and 12-week treatment phase) (Fig. 1). The washout phase was introduced to normalize the oral hygiene habits of participants as also suggested by Sreenivasan et al. [35]. Participants who met the inclusion and exclusion criteria were recruited in the 2-week washout phase, and were instructed to brush their teeth with Bass brushing technique twice daily for 3 min using Crest herbal crystal toothpaste and a Crest triple care toothbrush (P&G Technology Co., Ltd., Beijing, China). Participants were asked not to use antibiotics/hormonal drug and have periodontal therapy during the washout phase. These participants were re-screened according to the inclusion and exclusion criteria at baseline, and then were enrolled in the 12-week treatment phase.

The sample size was calculated using G*Power 3.1 software and was based on the primary data of gingival bleeding. Based on inequality and two independent groups (Fisher’s exact test), the sample size was determined to be 60, with an 0.05 alpha level (type II error) and 90% power (type I error). Considering a 15% dropout rate, 70 participants were eventually enrolled in this study.

The participants were randomly allocated to two groups (*n* = 35/group) using a computer-generated randomization list. The control group only brushed their teeth with Bass brushing technique twice daily for 3 min using abovementioned toothbrush and toothpaste, but the experimental group was additionally instructed to use a Bixdo P50 water flosser (Bixdo SH Healthcare Technology Co., Ltd., Shanghai, China) to irrigate all the teeth in the oral cavity with water for 3 min immediately after toothbrushing. Participants were recalled at 4, 8, and 12 weeks, and their modified gingival index, sulcus bleeding index, bleeding on probing (BOP), dental plaque index, and oral malodor values were determined. Supragingival plaque samples were also collected. Participants were asked to refrain from brushing, flossing, eating, and drinking anything other than water for 4 h prior to sample collection visits. The names and groups of the participants were covered for blinding to those collecting samples or clinical assessments. Over the experimental period, participants received weekly oral hygiene instruction and supervised plaque removal using the devices assigned for each group.

### Periodontal examination and supragingival plaque collection

Periodontal examination was performed by an operator, previously trained until reaching a low intra-operator variability, using an UNC-15 periodontal probe with a controlled (ca. 0.25 N) force. The gingival sulcus depth (distance from the gingival margin to the bottom of the gingival sulcus, evaluated at each tooth surface, for all teeth), modified Loe–Silness gingival index (assessment of gingival color, texture, and bleeding tendency, evaluated at four specific sites of each tooth), sulcus bleeding index (assessment of gingival bleeding, with a score of 0–5 per site), BOP (recorded based on the presence or absence of bleeding up to 20 s after probing at the experimental sites), and dental plaque index (evaluation of the presence of plaque, with a score of 0–5 per site) were measured [36]. After drying saliva off the collection site, supragingival plaque samples were collected from facial and lingual sites of all six Ramfjord teeth of each subject, by using a sterile curette. Scraped plaque was immediately transferred to and dispersed in 1 × phosphate-buffered saline in a sterile microcentrifuge tube. The plaque sample was divided into three parts after vibration and stored at − 80 °C until analysis.

### Oral malodor measurement using a halimeter

Oral malodor values were measured using a halimeter (RH-17 K; Interscan Co., Chatsworth, CA). Subjects refrained from any oral activity, such as food intake, for at least 2 h pre-measurement. The subjects were instructed to close their mouths for 3 min (using only nasal breathing) before every measurement, followed by placing a straw, connected to the halimeter, 4-cm deep into their mouth. The measurement was performed automatically, and the mean value of three consecutive measurements was used for analysis [37].

### 16S rRNA sequencing

Barcoded 16S rRNA amplicon (V3–V4 regions) sequencing was performed using Illumina MiSeq technology (Personalbio, Shanghai, China) and primers F (5′-ACTCCTACGGGAGGCAGCA-3′) and R (5′-TCGGACTACHVGGGTWTCTAAT-3′). A unique 12-mer tag for each DNA sample was added to the 5′-end of both primers to allow pooling of multiple samples in one run. The PCR products were visualized on a 3% agarose gel, gel purified, quantified with a Pico-Green kit, pooled in an equimolar ratio, assessed using an Agilent BioAnalyzer 2100 (Invitrogen, Carlsbad, CA), and sequenced. Sequences were trimmed using Trimmomatic 2 based on a quality score of 20, and pair-end reads were merged into longer reads using FLASH 3. Unqualified sequences were removed if they were too short or if they contained ambiguous residues. Operational taxonomic units (OTUs) were clustered using Usearch (version 7.1, http://drive5.com/uparse/) at a 97% similarity level. The final OTUs were generated based on clustering results. The raw sequencing data were deposited in the public database Sequence Read Archive (http://www.ncbi.nlm.nih.gov/Traces/sra) with accession no. PRJNA861384.

Before bioinformatic analysis, sequencing reads of all samples were standardized by rarefying OTU tables to the minimum read number. Analyses were performed using the online Majorbio Cloud Platform (www.majorbio.com) [38]. The Kruskal–Wallis *H* and Wilcoxon rank-sum tests were used to compare differences in taxa. Alpha diversity was calculated in terms of Chao, Shannon, and Simpson indices and was compared using the Wilcoxon rank-sum test. Beta diversity was assessed by principal coordinate analysis (PCoA) or non-metric multidimensional scaling (NMDS) analysis using weighted-unifrac distance and Adonis with 999 permutations. Analysis of similarity values and heatmaps were constructed using R (version 3.3.1; https://www.r-project.org/) “vegan” (version 2.4–3) package. Linear discriminant analysis (LDA) of the effect size (LEfSe) was performed to identify the significant taxa that most likely explained the differences between groups, with a threshold LDA score of 2. Bugbase was used for the predictions of the functional profile of a microbial community based on 16S rDNA sequence data. BugBase is able to predict seven phenotype types, including gram-positive, gram-negative, biofilm forming, pathogenic, mobile element containing, oxygen utilizing, and oxidative stress tolerant. A *p* value of < 0.05 was considered statistically significant in the current study.

### Bacterial quantification

Quantitative polymerase chain reaction (qPCR) was used to quantify dental plaque bacteria. qPCR amplification was performed using the CFX96 system (Bio-Rad, Hercules, CA). The 25-μl reaction mixture contained the SYBR reaction mix (TaKaRa, Kusatsu, Japan), template DNA (100 ng), and forward and reverse primers (500 nM each). Thermal cycling conditions were as follows: initial denaturation at 95 °C for 30 s, followed by 40 cycles each consisting of 95 °C for 15 s, and 60 °C for 30 s. Threshold cycle (CT) values were determined, and the relative abundance was calculated based on the 2^−ΔΔCT^ method. *Porphyromonas gingivalis*, *Fusobacterium nucleatum*, *Actinobacillus actinomycetemcomitans*, and *Prevotella intermedia* were quantified using primers listed in Table S1. Each sample was examined in triplicate.

### Statistical analysis

Statistical analysis of data other than 16S rRNA sequencing was performed using SPSS software (version 16.0; SPSS Inc., Chicago, IL) and GraphPad Prism 8 (GraphPad Software Inc., La Jolla, CA). Categorical variables are presented as natural frequencies, and continuous variables as mean ± standard deviation. The clinical characteristics of the participants were analyzed using Kruskal–Wallis (with Dunn–Bonferroni post hoc) or chi-squared tests. Within-group and between-group differences in gingival, sulcus bleeding, and dental plaque indices, BOP%, and oral malodor values were analyzed by repeated measure (RM) ANOVA, followed by Bonferroni post hoc tests and Student’s *t* tests, respectively. Other data were analyzed using one-way ANOVA, followed by Tukey’s test or the Student–Newman–Keuls test to compare all pairs of groups. Data were considered significantly different if the two-tailed* p* value was < 0.05.

## Results

### Clinical characteristics of the study participants

This study enrolled 70 gingivitis participants, of which seven dropped out, who missed the recall time due to closed management amid COVID-19 outbreak. Thirty-three control and 30 experimental participants attended all recall visits. The baseline clinical characteristics are shown in Table 1. Demographics, gingival, and halitosis parameters were similar between the groups (*p* > 0.05). No adverse reaction including gingival recession reported during the 12-week experimental period in terms of water flossing.

**Table 1:** Clinical characteristics of the participants at baseline

	Control group (*n* = 33)	Water-flossing group (*n* = 30)	*p* value
Age (years)	34.20 ± 11.99	31.05 ± 10.00	0.265
Gender (M/F)	11/22	14/16	0.313
Ethnicity (Han/other)	33/0	29/1	0.476
BMI (kg/m^2^)	22.82 ± 3.35	22.60 ± 3.74	0.798
No. of teeth	28.85 ± 1.97	28.63 ± 1.63	0.629
Past medical history (no/yes)	30/3	24/6	0.289
Gingival sulcus depth	1.51 ± 0.27	1.41 ± 0.22	0.115
Gingival index	1.26 ± 0.17	1.23 ± 0.16	0.493
Sulcus bleeding index	1.70 ± 0.44	1.63 ± 0.41	0.554
Bleeding on probing (%)	48.23 ± 23.91	43.61 ± 21.85	0.428
Dental plaque index	2.46 ± 0.61	2.60 ± 0.51	0.335
Oral malodor values	177.14 ± 96.10	201.65 ± 119.40	0.371

Continuous variables are presented as mean ± standard deviation. Categorical variables are presented as natural frequencies

*M* male, *F* female, *BMI* body mass index

### Effects of water flossing on clinical parameters

The effects of water flossing on periodontal parameters, dental plaque index, and oral malodor were assessed. Two-way RM-ANOVA identified time and group as statistically significant factors (*p* < 0.05) in the gingival index, sulcus bleeding index, and BOP% (Table 2). These indices improved significantly in both groups at all observation time points (weeks 4, 8, 12) compared with baseline (*p* < 0.05). However, the improvement in the control group slowed after 4 weeks. Gingival index and sulcus bleeding index at weeks 8 and 12 were significantly better in the experimental than in the control group, and BOP% improved significantly in the experimental group at weeks 8 as compared with the control group (Table 2). These results suggest that water flossing had a good clinical effect on managing gingival bleeding and inflammation.

**Table 2:** Two-way repeated-measure ANOVA analysis results of the clinical parameters

Values	Time point	Control group (*n* = 33)	Water-flossing group (*n* = 30)	*p* value (RM-ANOVA analysis)	
				Time	Group
Gingival index	W0	1.26 ± 0.17	1.23 ± 0.16	< 0.0001	0.0180
	W4	1.06 ± 0.30^#^	1.02 ± 0.21^$^		
	W8	1.07 ± 0.27^#^	0.89 ± 0.26^$**^		
	W12	0.99 ± 0.22^#^	0.83 ± 0.28^$*^		
Sulcus bleeding index	W0	1.70 ± 0.44	1.63 ± 0.41	< 0.0001	0.0234
	W4	1.36 ± 0.45^#^	1.25 ± 0.37^$^		
	W8	1.35 ± 0.41^#^	1.11 ± 0.36^$*^		
	W12	1.28 ± 0.31^#^	1.09 ± 0.38^$*^		
Bleeding on probing (%)	W0	48.23 ± 23.91	43.61 ± 21.85	< 0.0001	0.0099
	W4	31.82 ± 25.47^#^	24.72 ± 19.27^$^		
	W8	34.09 ± 23.51^#^	17.50 ± 16.43^$**^		
	W12	25.76 ± 20.13^#^	17.78 ± 16.20^$^		
Dental plaque index	W0	2.46 ± 0.61	2.60 ± 0.51	0.0316	0.5556
	W4	2.37 ± 0.64	2.45 ± 0.64		
	W8	2.36 ± 0.55	2.30 ± 0.52^$^		
	W12	2.29 ± 0.44	2.40 ± 0.52		
Oral malodor values	W0	177.14 ± 96.10	201.65 ± 119.40	0.1392	0.3708
	W4	146.61 ± 90.20	183.60 ± 100.80		
	W8	149.14 ± 69.42	186.12 ± 113.33		
	W12	174.49 ± 137.89	151.23 ± 77.02^$^		

*W* week, *RM-ANOVA analysis* repeated-measure ANOVA analysis

^#^Significant differences compared with the baseline in the control group

^$^Significant differences compared with the baseline in the experimental group

^*^Between-group differences at the same time point

*p < 0.05, **p < 0.01

Two-way RM-ANOVA of the dental plaque index identified time as a statistically significant factor (*p* < 0.05), with significant differences among time points (Table 2). However, the control and experimental groups did not differ at any time point (Table 2). There was no significant difference in the oral malodor values between groups after the use of the respective oral hygiene regimens (Table 2). However, the value was significantly decreased after 12-week water flossing as compared with that at baseline in the experimental group (Table 2).

### Effects of water flossing on dental plaque microbiota

The 16S rRNA sequencing data are detailed in the supplementary information (Fig. S1). In total, 20.71 million high-quality 16S rRNA sequences were generated from the supragingival plaque samples of 33 control and 30 experimental participants at 0, 4, 8, and 12 weeks (length range 408–480 bp, average length 464.36 bp). After subsampling each sample to equal sequencing depth and clustering, 6442 OTUs with 97% identity were obtained. Good’s coverage for the observed OTUs was 99.72 ± 0.09%, and the rarefaction curves showed clear asymptotes, which together indicated a near-complete sampling of the community.

**Fig. 2:**
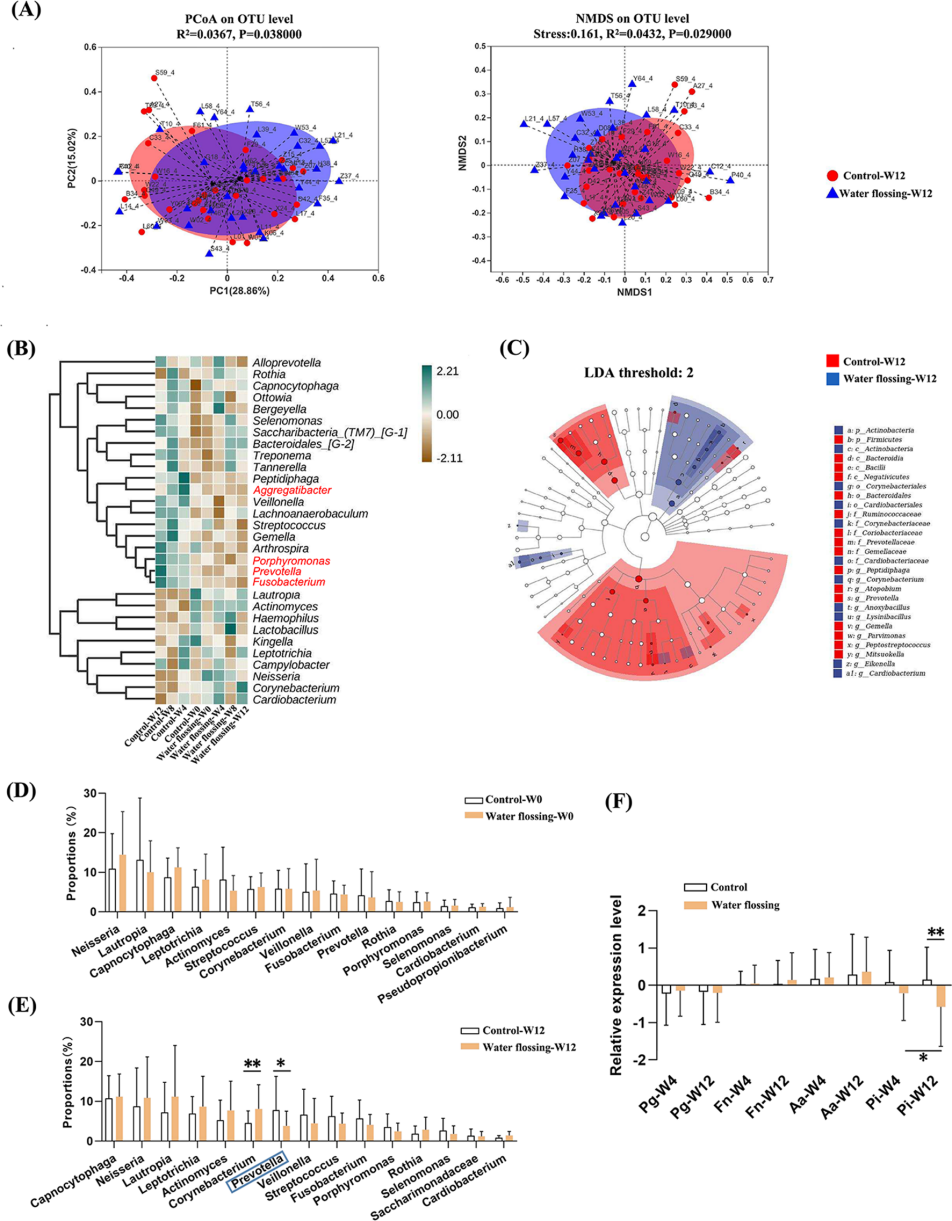
Comparison of the microbiota community structure and composition between the control and water flossing group. **A** Principal coordinate analysis (PCoA) and non-metric multidimensional scaling (NMDS) analysis of the dental plaque samples from control and experimental groups at week 12. **B** Heatmap analysis of the top 30 abundant bacterial taxa at genus level. **C** Linear discriminant analysis of the effect size (LEfSe) analysis at week 12. **D** Intergroup difference analysis of the top 15 abundant bacterial taxa at genus level at baseline. **E** Intergroup difference analysis of the top 15 abundant bacterial taxa at genus level at week 12. **F** Relative quantification of *Porphyromonas gingivalis*, *Fusobacterium nucleatum*, *Actinobacillus actinomycetemcomitans*, and *Prevotella intermedia* at weeks 4 and 12 as compared to baseline. *p < 0.05, **p < 0.01

The *α* and *β* diversity of the baseline dental plaque samples showed no significant difference between the two groups, suggesting that the baseline dental plaque microbiota structure of the two groups was comparable (Fig. S2A and B). Although the PCoA analysis showed no significant difference in the microbiota community structure between the groups, the *p* value approximated 0.05 (*p* = 0.056). NMDS analysis revealed a significant difference between the groups (*p* = 0.012) (Fig. S2C). PCoA and NMDS analyses demonstrated significant differences between the control and experimental groups at week 12 (PCoA: *p* = 0.038, NMDS: *p* = 0.029), and samples could be clearly distinguished and clustered into two groups (Fig. 2A). These results indicate that, after 12 weeks, the dental plaque microbiota of the two groups were distinct, suggesting that water flossing changed the microbial structure.

Further analysis of the top 30 abundant bacterial taxa revealed genus-level differences between the control and water-flossing groups (Fig. 2B). The abundance of *Aggregatibacter*, *Porphyromonas*, *Prevotella*, and *Fusobacterium* increased over time in the control, but not in the experimental group (Fig. 2B). There was no significant difference in the top 15 most abundant bacterial taxa between groups at baseline (Fig. 2D). However, after 12 weeks, the water-flossing group exhibited significantly lower *Prevotella* and higher *Corynebacterium* genus as compared with the control group (Fig. 2C, E). qPCR showed no difference in the relative abundance of *Porphyromonas gingivalis*, *F. nucleatum*, and *A. actinomycetemcomitans* between the groups at weeks 4 or 12, as compared to baseline, but the abundance of *Prevotella intermedia* in the experimental group was significantly lower at week 12 than week 4 or that in the control group at week 12 (Fig. 2F).

**Fig. 3:**
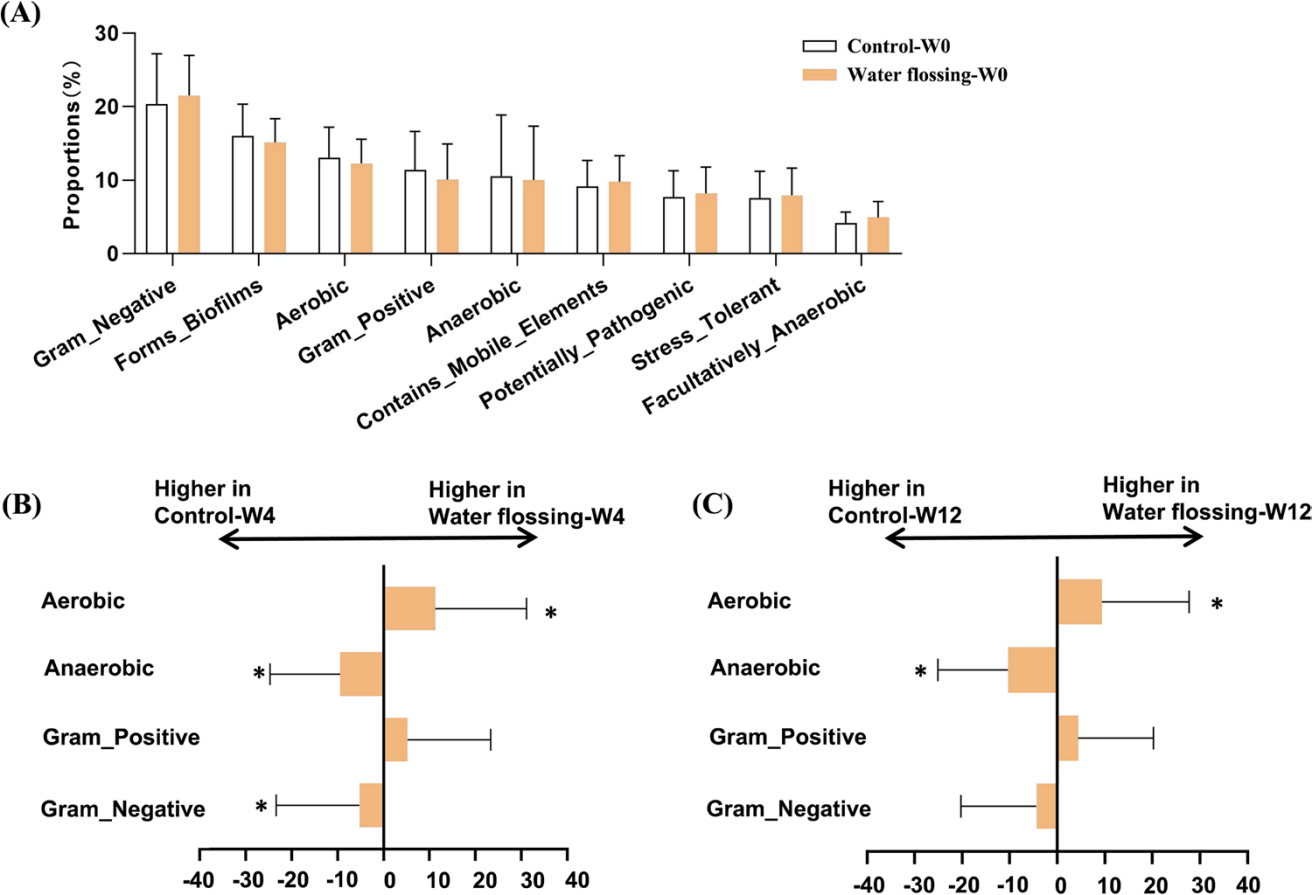
Phenotype prediction of the dental plaque community. **A**. BugBase phenotype prediction of the control and experimental groups at baseline. **B**. BugBase phenotype prediction of the control and experimental groups at week 4. **C**. BugBase phenotype prediction of the control and experimental group at week 12. *p < 0.05, **p < 0.01

The BugBase phenotype prediction showed no significant difference in the seven metabolic phenotypes between the control and experimental groups at baseline (Fig. 3A). At week 4, the water-flossing group exhibited significantly higher aerobic phenotype, while the control group exhibited significantly higher anaerobic and gram-negative phenotypes (Fig. 3B). At week 12, the water-flossing group still exhibited significantly higher aerobic phenotype, while the control group exhibited significantly higher anaerobic phenotype (Fig. 3C). These data further suggest that water flossing may benefit periodontal health by altering the microbial composition and reducing the virulence of the dental plaque.

## Discussion

Gingivitis is a typical plaque-related oral diseases, and plaque control has been proved to be an effective way to prevent and treat this disease. Accumulating evidence has demonstrated that water flossing can ameliorate gingival inflammation by removing dental plaque [25, 39, 40]. Although relatively easy and safe to apply as a daily oral hygiene instruction, the ecological impact of water flossing on oral microbiota after long-term use has yet to be investigated. In addition, as oral malodor is usually accompanied with periodontal diseases including gingivitis and periodontitis, whether or not water flossing can benefit oral malodor control still needs clinical validation. Here, we performed a prospective clinical trial to investigate the effects of water flossing on plaque accumulation, gingival inflammation, and halitosis, as well as the ecological impact on supragingival plaque microbiota in a duration of 12-week application. We demonstrate that water flossing is an effective adjuvant to control gingival inflammation.

Water flossing promotes gingival health by facilitating removal of dental plaque. Here, we found that both water-flossing and toothbrushing groups exhibited ameliorated gingival inflammation as reflected by gingival index, sulcus bleeding index, and BOP%. More importantly, the gingival and sulcus bleeding indices in the water-flossing group were lower than those in toothbrushing control at 8 and 12 weeks, indicating that adjunctive application of water flossing to toothbrushing can better promote periodontal health. Consistently, several randomized controlled trials found that the addition of a water flosser to toothbrush could significantly reduce the BOP, gingival index, and plaque index at 4-week compared with the toothbrush alone [39, 41]. Of note, although both groups exhibited significantly reduced plaque accumulating as reflected by dental plaque index, no significant between-group difference in dental plaque index was observed in this study. Studies also demonstrated that although use of water flosser plus manual toothbrushing significantly reduced the plaque index at 8-week compared with that at baseline, no between-group difference was achieved [42, 43]. The possible explanation is that dental plaque may have reformed overnight as the participants were instructed not to brush, floss, or eat for 4 h prior to sample collection. Moreover, the participants received good oral hygiene instruction and supervision during the study, and this may overwhelm the differences in dental plaque accumulation between the adjunctive water-flossing group and toothbrushing control group.

As water flossing is a well-recognized oral hygiene technique via active agitation and removal of supragingival dental plaque, whether its daily use may pose long-term disturbance on oral microecology is worth clinical validation. Here, we observed an altered microbial structure and composition after 12-week use of the respective oral hygiene regimens. More importantly, we also observed that periodontal anaerobes, such as *P. intermedia*, was depleted in the water-flossing group, while this anaerobe increased in toothbrushing control during the 12-week study. *P. intermedia*, known as “orange complex” species, is associated with gingivitis and periodontitis [44]. *P. intermedia* can stimulate the release of proteinases, matrix metalloproteinases, and proinflammatory cytokines, and favors the colonization of red complex species (i.e., *P. gingivalis*, *Treponema denticola*, *Tannerella forsythia*), triggering dental plaque dysbiosis and contributing to gingival inflammation, and eventually periodontitis [35, 44]. Consistently, we found that the oral microbiota of individuals using water flossing was prone to an aerobic phenotype, while the oral microbiota of the toothbrushing control was characterized with anaerobic and gram-negative phenotypes. Periodontal pathogens are mostly gram-negative anaerobes, enrichment of which can shift the composition and structure of the microbial community, leading to a breakdown of the normal homeostatic state [45]. It is speculated that water flossing may increase the presence of oxygen in the dental plaque, and thus favor the outgrowth of aerobes over anaerobic species. In addition, we also found that *Corynebacterium* genus was increased after 12-week use of water flosser. *Corynebacterium* genus is an important bridge organism in dental plaque, which is usually enriched in the periodontal healthy group, and is negatively associated with probe depth in patients with chronic periodontitis [46, 47]. Our findings indicate that water flossing may benefit gingival health by altering microbial composition, preventing overgrowth of oral pathobionts, and promoting anerobic phenotype of the dental plaque.

In addition to gingival inflammation, oral malodor, which is mainly derived from the metabolic production of volatile sulfur compounds (VSCs) by periodontal anaerobes, is usually accompanied with periodontal conditions such as periodontitis and gingivitis [48, 49]. Although there still lacks clinical evidence, it is conceivable that water flossing may help reduce oral malodor by alleviating gingival inflammation and reducing oral anaerobes. The current study measured the halimeter score of participants during the 12-week use of water flosser. We found that participants in the water flossing group had significantly lower oral malodor values at week 12 as compared to its baseline. This may be accredited to the reduced anaerobes and relative aerobic phenotype of dental plaque in the water flossing group.

In conclusion, the current study demonstrates that water flossing can effectively alleviate gingival inflammation and reduce oral malodor, possibly by depleting oral anaerobes and altering the oral microbiota to a more aerobic phenotype.

## Supplementary Information

Below is the link to the electronic supplementary material.

Supplementary file1 (DOCX 5382 KB)

## Data Availability

The data are available upon request from the corresponding author. The 16S rRNA sequencing raw data were deposited in the public database Sequence Read Archive (http://www.ncbi.nlm.nih.gov/Traces/sra) under accession number PRJNA861384.
