# Supplementary material for: Effects of water flossing on gingival inflammation and supragingival plaque microbiota: a 12-week randomized controlled trial
Source: Clin Oral Investig. 2023 May 25;27(8):4567–77. doi: 10.1007/s00784-023-05081-4 (PMC10212231; doi:10.1007/s00784-023-05081-4)
Supplement: Supplementary file 1 — Supplementary file1 (DOCX 5382 KB) [file 784_2023_5081_MOESM1_ESM.docx]

# **Effects of water flossing on gingival inflammation and supragingival plaque microbiota: a 12-week randomized controlled trial**

**Xin Xu^1, 2^, Yishan Zhou^1, 2^, Chengcheng Liu^1, 3^, Lei Zhao^1, 3^, Ling Zhang^1, 2^, Haolai Li^1, 4^, Yi Li^5^, Xingqun Cheng^1, 6*^**

^1^The State Key Laboratory of Oral Diseases & National Clinical Research Center for Oral Diseases, West China Hospital of Stomatology, Sichuan University, Chengdu, Sichuan, China

^2^Department of Cariology and Endodontics, West China Hospital of Stomatology, Sichuan University, Chengdu, Sichuan, China

^3^Department of Periodontology, West China Hospital of Stomatology, Sichuan University, Chengdu, Sichuan, China

^4^State Institute of Drug/Medical Device Clinical Trial, West China Hospital of Stomatology, Sichuan University, Chengdu, Sichuan, China

^5^Bixdo (SH) Healthcare Technology Co., Ltd., Shanghai, China

^6^Department of Geriatric Dentistry, West China Hospital of Stomatology, Sichuan University, Chengdu, Sichuan, China

**^*^Correspondence author:**

Xingqun Cheng; E-mail: [chengxq2007@163.com](mailto:chengxq2007@163.com)

Address: No. 14, 3rd section of Renmin South Road, Chengdu, Sichuan, China, 610041

**Supplementary materials**

1. **FIGURES**

**
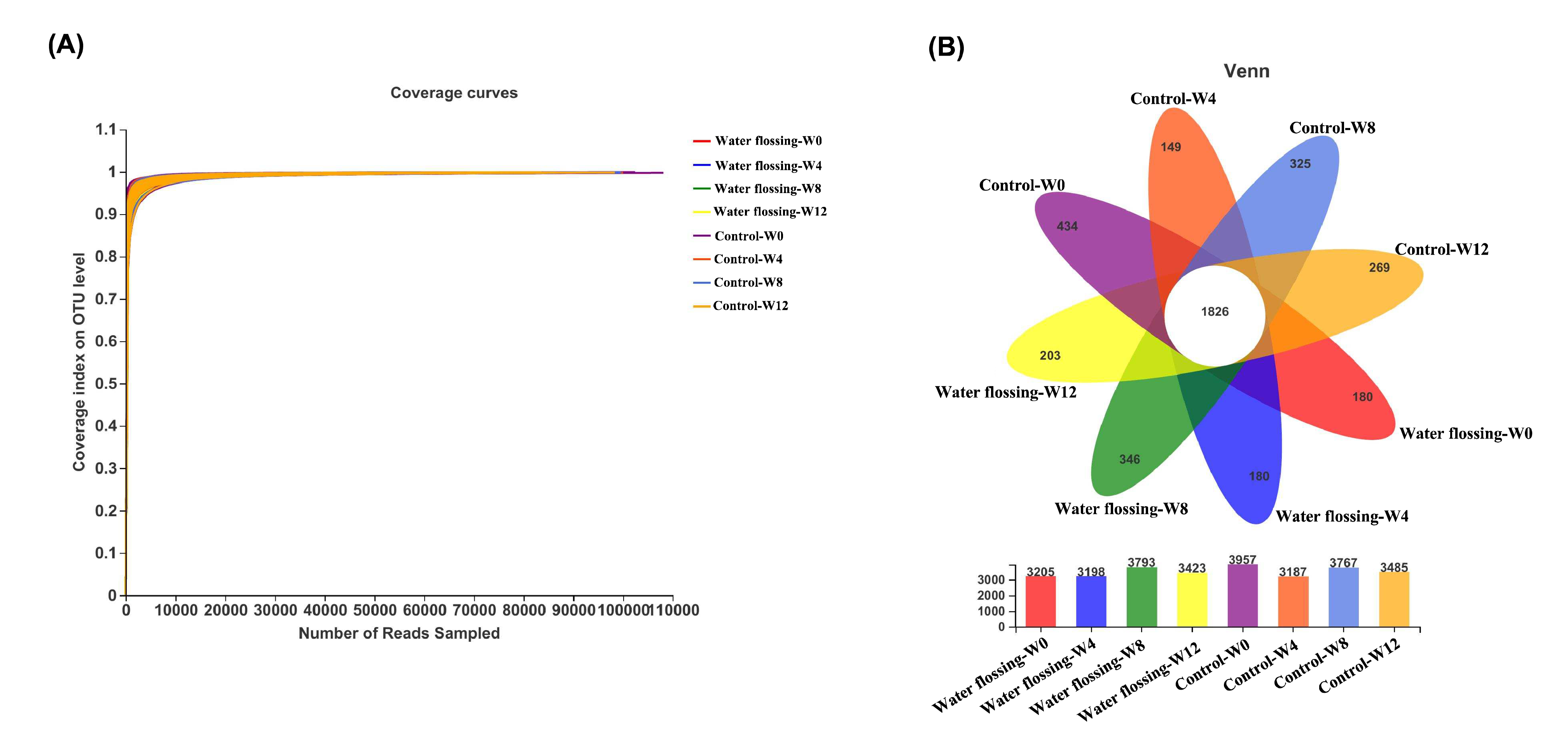
Fig. S1** Rarefaction curves and Venn diagram.

**
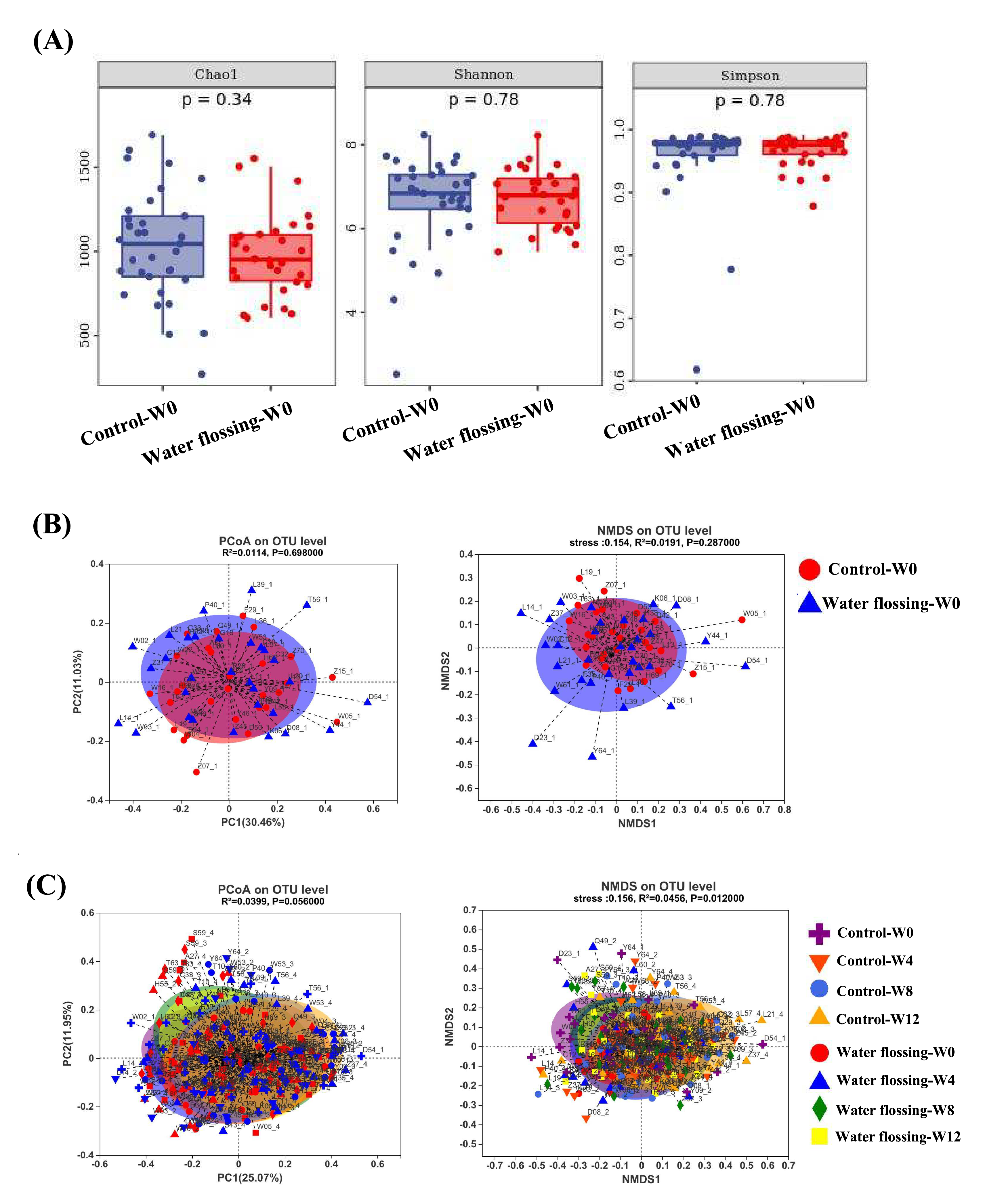
**

**Fig. S2** The α and β diversity analyses of the baseline dental plaque samples. A. The α diversity of dental plaque microbiota. B. Principal coordinate analysis (PCoA) and non-metric multidimensional scaling (NMDS) analysis of dental plaque microbiota. W0 indicates week 0 (baseline). C. PCoA and NMDS analysis of the dental plaque samples from the control and experimental groups at weeks 0, 4, 8, and 12. W, week.

1. **TABLE**

**Table S1. Primers used in the study.**

| **Primer name** | **Sequence (5’-3’)** | **Reference** |
| --- | --- | --- |
| **Pg** | F: TACCCATCGTCGCCTTGGT  R: CGGACTAAAACCGCATACACTTTG | Di Murro et al., 2021 |
| **Fn** | F: CGCAGAAGGTGAAAGTCCTGTAT  R: TGGTCCTCACTGATTCACACAGA | Di Murro et al., 2021 |
| **Pi** | F: CGTGGACCAAAGATTCATCGGTGGA  R: CCGCTTTACTCCCCAACAAA | Di Murro et al., 2021 |
| **Aa** | F: CTTACCTACTCTTGACATCCGAA  R: ATGCAGCACCTGTCTCAAAGC | Di Murro et al., 2021 |

Abbreviations: Pg, *Porphyromonas gingivalis*; Fn, *Fusobacterium nucleatum*; Pi, *Prevotella intermedia*; Aa, *Aggregatibacter actinomycetemcomitans*; F, Forward; R, Reverse.

1. **REFERENCES**

Di Murro B, Moretti M, De Smaele E, et al (2021) Microbiological Profiles of Dental Implants in Metabolic Syndrome Patients: A Case-Control Study. Antibiotics (Basel) 10(4):452. https://doi.org/10.3390/antibiotics10040452
